# Effects of midazolam on cardiovascular responses and isoflurane requirement during elective ovariohysterectomy in dogs

**DOI:** 10.1186/s13620-018-0136-y

**Published:** 2018-12-17

**Authors:** Josephine Kropf, J.M. Lynne Hughes

**Affiliations:** 1 Anaesthesia Department, Small Animal Hospital, School of Veterinary Medicine, University of Glasgow, 464 Bearsden Road, Glasgow, G61 1QH UK; 2 Veterinary Anaesthesia, UCD Veterinary Hospital, UCD School of Veterinary Medicine, University College Dublin, DO4 W6F6 Dublin, Ireland

**Keywords:** Canine, Ovariohysterectomy, Midazolam, Cardiovascular parameters, Isoflurane-sparing

## Abstract

**Background:**

A prospective, randomized, placebo-controlled, blinded clinical study was conducted to determine whether a single dose of midazolam affects the cardiovascular response to surgical manipulation of the ovaries during elective ovariohysterectomy. Thirty-nine client-owned dogs undergoing elective ovariohysterectomy were recruited. After scoring cage demeanour, dogs were premedicated with acepromazine (0.03 mg kg^-1^) and pethidine (3 mg kg^-1^) intramuscularly into the quadriceps muscle and 20 min later sedation was scored. Anaesthesia was induced with propofol intravenously (IV) to effect. The study treatment (group M: midazolam (0.25 mg kg^-1^); or group P: placebo (Hartmann’s solution) (0.125 ml kg^-1^)) was administered IV before the intra-operative manipulation of the first ovary. Anaesthesia was maintained with isoflurane in oxygen. Morphine (0.3 mg kg^-1^ IV) was administered prior to the start of surgery. The vaporizer setting was adjusted according to the depth of anaesthesia. If an end-tidal isoflurane concentration (FE’Iso) above 1.6% was required additional analgesia was provided with fentanyl (2 μg kg^-1^). Dogs received meloxicam (0.2 mg kg^-1^ IV) at the end of procedure. Heart rate, mean arterial blood pressure, respiratory rate and end-tidal partial pressure of carbon dioxide as well as FE’Iso were recorded and analysed.

**Results:**

A statistical significant difference between groups was detected in FE’Iso, with group M requiring a significantly lower FE’Iso than group P (14.3%) after administration of midazolam. No differences between groups was shown for percentage change in heart rate and mean arterial blood pressure, or end-tidal carbon dioxide and requirement for mechanical ventilation, or rescue analgesia. There was no statistically significant difference in the incidence of complications in group M and P. Group M received significantly more succinylated gelatin solution pre-administration of midazolam than group P, but no differences in fluid administration post-administration of the study treatment (midazolam/placebo) were detected. No statistical significant difference was demonstrated for the use of anticholinergic agents, dobutamine or noradrenaline.

**Conclusion:**

No significant effect on cardiovascular parameters could be observed with administration of midazolam, but a modest (14.3%) isoflurane-sparing effect was detected.

## Background

Ovariohysterectomy of the bitch is a common surgical procedure performed in veterinary medicine [1–3] and it is quite often the first surgery veterinary medicine students and recent graduates will be asked to perform [1, 4].

The canine ovary is richly innervated by sensory, sympathetic and parasympathetic nerve fibres, that travel alongside the ovarian artery and vein inside the ovarian pedicle [5–8]. Traction on the suspensory ligament and clamping of the ovarian pedicle generate a particularly painful stimulus during the ovariohysterectomy procedure [9]. The mesovarial injection of local anaesthetic resulted in a significantly lower pain response in cats, horses and humans [8, 10, 11]. In contrast, mesovarial injection of lidocaine in dogs undergoing ovariohysterectomy did not decrease isoflurane requirements, nor lessen the autonomic response to surgical manipulation [9]. Other methods may have to be considered to attenuate the nociceptive response to the surgical manipulation of the ovarian pedicle, and veterinarians working in general practice have anecdotally suggested that the administration of benzodiazepines may have such an attenuating effect.

Intra-operative detection of pain relies on the observation and interpretation of autonomic changes, especially cardiovascular and respiratory responses [12–14]. Anaesthesia and analgesia are intrinsically linked and autonomic parameters can both be an indicator of changes in the nociception – anti-nociception balance and/or changes in depth of anaesthesia [13, 14].

Midazolam is an imidazobenzdiazepine that acts as an agonist on the GABA_A_ receptor. Similar to other benzodiazepine agonists it induces anticonvulsant, anxiolytic, sedative/hypnotic, amnesic and centrally mediated muscle relaxant effects [15]. Systemically administered it is not thought to be analgesic [16]. It was shown to cause minimal cardiovascular changes at clinically relevant doses [17] but is associated with a dose-related centrally mediated respiratory depression [18].

The primary objective of the study was to determine the effect of midazolam on the cardiovascular response to the surgical manipulation of the ovaries during elective ovariohysterectomy. A secondary objective was to determine the effect of midazolam on the isoflurane concentration required to maintain a surgical plane of anaesthesia.

Our null hypothesis was that administration of a single bolus of midazolam at a dose of 0.25 mg kg^-1^ would not exert a significant effect on the cardiovascular parameters heart rate (HR) and mean arterial blood pressure (MAP) in healthy dogs undergoing elective ovariohysterectomy at a tertiary referral university teaching hospital. Our secondary null hypothesis was that the administration of midazolam would have no isoflurane-sparing effect during surgical manipulation.

## Methods

### Animals

The conducted study was approved by the University College Dublin (UCD) Office of Animal Research Ethics (approval number: AREC-E-16-27-Hughes) and signed, informed owner consent was obtained for all patients included.

For the purpose of this study, 39 healthy female dogs, presented for elective ovariohysterectomy at the UCD Veterinary Hospital, were prospectively recruited. Both privately owned dogs and shelter dogs were included in the study. All were assigned an American Society of Anesthesiologists (ASA) physical status grade 1. Patients weighing less than 5 kg, paediatric (≤ 12 weeks) and geriatric patients (dogs in the last 25% of their predicted life span) and patients assigned an ASA physical status grade other than 1 were excluded.

Dogs were randomised with an online list randomizer [19] to receive either midazolam (Hypnovel® 10 mg ml^-1^, Roche Products (UK) Ltd.; 0.25 mg kg^-1^) (group M) or an equivalent volume of placebo (Hartmann’s Lactated Ringer’s solution; B. Braun Melsungen AG, Germany; 0.125 ml kg^-1^) (group P) intravenously (IV) five minutes prior to the commencement of surgical removal of the first ovary.

### Experimental protocol

Prior to any interaction with the patient, the primary investigator observed the animal from a distance in the kennel and assigned a cage demeanour score with the help of a simple descriptive scale adapted from Ferreira et al. (2015) (Table 1) [20]. A clinical examination and pre-anaesthetic assessment, but no blood testing, was performed and an ASA physical status grade was assigned to the patient.

Dogs were premedicated with acepromazine (Calmivet® 5 mg ml^-1^, Vetoquinol (France); 30 μg kg^-1^) and pethidine (Pethidine Hydrochloride 50 mg ml^-1^, Mercury Pharmaceuticals (Ireland) Ltd.; 3 mg kg^-1^) by intramuscular injection into the quadriceps muscle. A simple descriptive sedation score (Table 2) [21] was assigned 20 min after premedication shortly followed by the placement of an appropriately sized IV cannula in one of the cephalic veins. A tight-fitting oxygen mask was then applied over the nose of the dog with an oxygen flow of 5 L min^-1^ supplied by the anaesthetic machine.

All dogs were anaesthetized by the main investigator, who was unaware of the group allocation (midazolam or placebo). Following IV cannula placement, anaesthesia was induced with propofol (Vetofol, 10 mg ml^-1^, Norbrook Laboratories (Ireland) Ltd.) IV to effect until jaw tone was relaxed and endotracheal intubation was possible. The trachea of all dogs was intubated with a cuffed endotracheal tube of appropriate size and animals were connected to an anaesthetic machine via a circle breathing system.

Anaesthesia was maintained with isoflurane (Iso-Vet 1000 mg g^-1^, Chanelle, Piramal Healthcare UK) vaporised in oxygen with an oxygen flow set at 100 ml kg^− 1^ min^− 1^ for the first 15 min, and then reduced to 10 ml kg^-1^ min^-1^. All dogs were allowed to breathe spontaneously throughout the procedure. Intermittent positive pressure ventilation was initiated only if end-tidal partial pressure of carbon dioxide (PE’CO_2_) values exceeded 6.9 kPa or a period of apnoea with intermittent manually delivered breaths longer than five minutes occurred.

For the duration of the anaesthetic, intravenous fluid support was provided with Hartmann’s solution (Hartmann’s Lactated Ringer’s solution; B. Braun Melsungen AG, Germany) at a rate of 10 ml kg^-1^ h^-1^.

**Fig. 1:**
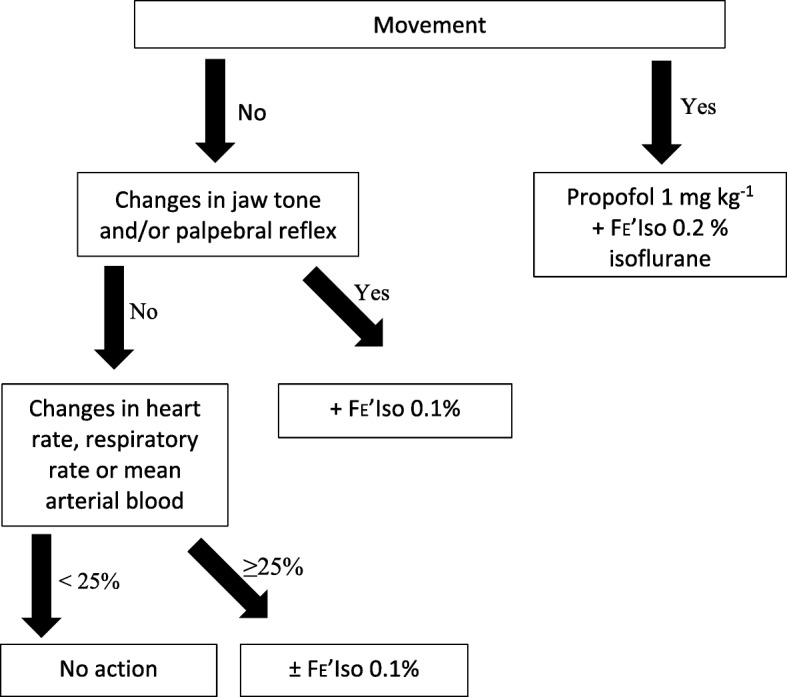
Flow chart for adjustment of anaesthetic depth in isoflurane anaesthetised healthy dogs undergoing ovariohysterectomy, adapted from Enderle et al., 2008 [75]. End-tidal isoflurane concentration, FE’Iso

At the beginning of anaesthesia, the vaporizer was initially set to achieve an end-tidal isoflurane concentration (FE’Iso) concentration of 1.1%. The dogs were continuously monitored with a manual assessment of muscle relaxation (jaw tone), eye position and palpebral reflex, and a multiparameter monitor (B40; GE Healthcare, USA). The vaporizer setting was subsequently adjusted to maintain or alter FE’Iso concentration according to the depth of anaesthesia. If a dog showed gross movement, an increase in jaw tone or a palpebral reflex, a propofol bolus (1 mg kg^-1^) was administered IV and FE’Iso was increased by 0.1–0.2%. If mean arterial blood pressure (MAP) or heart rate (HR) increased or decreased by more than 25% FE’Iso was also adjusted in 0.1% increments accordingly. If an FE’Iso above 1.6% was required, additional analgesia was provided with fentanyl (Sublimaze®, Janssen-Cilag Ltd., UK; 2 μg/kg) intravenously (Fig. 1).

**Table 1:** Cage dememeanour scoring system

Score	Description
1	Severely anxious and aggressive, vocalizing, no body tremors
2	Anxious and vocalizing, no body tremors
3	Anxious but responsive to external stimuli
4	Calm and responsive to external stimuli
5	Excited, happy and responsive to external stimuli

Cage demeanour scoring system used to assess 39 healthy female dogs admitted for elective ovariohysterectomy [20]

Monitored parameters were recorded every five minutes, starting at induction of anaesthesia, until the end of anaesthesia, defined as discontinuation of isoflurane. The parameters monitored included HR and electrocardiogram (ECG), respiratory rate *(f*_R_*),* PE’CO_2_ and FE’Iso, haemoglobin oxygen saturation and arterial blood pressure. During the preparation for surgery non-invasive oscillometric blood pressure was measured. A 22G arterial catheter was placed in the dorsal pedal artery and intraoperatively invasive MAP was measured using a pressure transducer zeroed to atmospheric pressure and placed at the level of the heart.

Intra-operative analgesia was provided to all dogs by the administration of morphine (Morphine Sulphate 10 mg ml^-1^, Mercury Pharmaceuticals (Ireland) Ltd.; 0.3 mg kg^-1^) slowly IV at the time of moving into the operating room.

Ovariohysterectomy was performed by final year veterinary students under direct supervision of a qualified veterinary surgeon. Once the first ovary was identified, the treatment (midazolam / placebo) was administered and the surgeons stopped all manipulation for five minutes to allow sufficient time for the onset of action of midazolam. Ovaries were removed using the triple clamp technique and double ligation. The time of administration of midazolam and the start and end of manipulation the ovaries was recorded. Manipulation of the ovaries was defined as stretching and tearing of the suspensory ligament, clamping and ligation of the ovarian pedicle, and removal of the ovaries.

For the purpose of the study, bradycardia was defined as HR below 80 min^-1^, tachycardia as a HR above 120 min^-1^, normotension as a MAP between 70 and 120 mmHg, normocapnia as an PE’CO_2_ between 4.0 and 7.0 kPa, and normothermia as a temperature of 37.0–39.0 °C. Bradycardic animals received either atropine (Atropine sulfate; Mercury Pharmaceuticals Ltd., Ireland; 20 μg kg^-1^) or glycopyrrolate (Glycopyrronium Bromide; Mercury Pharmaceuticals Ltd., Ireland; 5 μg kg^-1^) IV at the anaesthetist’s discretion. Hypotension was treated with a cascade of one to two boluses of crystalloid (Hartmann’s solution; 10 ml kg^-1^ over 15 min), followed by one to four boluses of colloid (succinylated gelatin solution; Gelofusine® 40 mg ml^-1^; B. Braun Melsungen AG, Germany; 5 ml kg^-1^ over 15 min), dobutamine constant rate infusion (CRI) (Dobutamine 12.5 mg ml^-1^; Mercury Pharmaceuticals Ltd., Ireland; 1–5 μg kg^-1^ min^-1^) and noradrenaline CRI (Noradrenaline; Hospira Enterprises B.V., The Netherlands; 0.1–1 μg kg^-1^ min^-1^). Apnoeic and persistently hypercapneic (i.e. > 5 min) patients were mechanically ventilated to achieve PE’CO_2_ values of 5.3 ± 0.5 kPa. Active warming was provided with a forced warm-air blanket (WarmTouch™ Patient Warming System, Nellcor (Medtronic), USA).

Following the end of surgery isoflurane was discontinued, 100% oxygen (100 ml kg^-1^ min^-1^) was administered for 10 min or until extubation, the fluid rate was decreased to 2.5 ml kg^-1^ h^-1^ and meloxicam (Loxicom, 5 mg ml^-1^, Norbrook Laboratories (Ireland) Ltd.; 0.2 mg kg^-1^) was administered IV.

Animals were extubated when starting to swallow or at the first sign of movement. During the recovery period oxygen (5 L min^-1^) was provided by face mask. All hypothermic animals were covered with an external heating device (forced warm-air blanket) until rectal temperature reached 37 °C. Animals vocalizing and/or thrashing in recovery were assessed for pain and dysphoria. Pain assessment was performed with the short-form Glasgow Composite Measure Pain Scale (CMPS-SF) [22]. To painful animals (CMPS-SF score ≥ 5/20 or 6/24) rescue analgesia was provided with morphine (0.2 mg kg^−1^) IV over five minutes. If the animal was not judged to be painful according to the CMPS-SF, post-anaesthetic dysphoria was assumed and acepromazine (2.5 μg kg^-1^) was administered IV.

A second dose of morphine (0.2 mg kg^-1^) was administered postoperatively three to four hours following the initial morphine dose and buprenorphine (Bupaq Multidose 0.3 mg ml^-1^ Injection for Dogs and Cats, Chanelle, Ireland; 15 μg kg^− 1^ IV q8h) was administered subsequently every 6–8 h for 24 h.

### Statistical analysis

Data analysis was performed using the statistical software SPSS 24. A power analysis using data from a pilot study, with eight dogs presented for ovariohysterectomy to the UCD Veterinary Hospital, was carried out and results indicated that a minimum of 16 animals per group was required to show a statistically significant difference between groups for a change in blood pressure from baseline of at least 25%. For the power analysis α was set at 0.05 and β at 0.2 with a power of 0.8. Normality was assessed using Shapiro-Wilk’s test and Normal Q-Q Plots. Data for HR, MAP, FE’Iso, *f*_R_ and PE’CO_2_ was partitioned into time periods (period 1: time in operating room until start of surgery; period 2: start of surgery to start of ovarian manipulation; period 3: manipulation of the ovaries; period 4: manipulation of the uterus; and period 5: abdominal closure) and mean values for each time period were calculated for statistical analysis. For HR and MAP, the percentage change from baseline (defined as the mean HR and mean MAP during period 1) was used for statistical analysis, rather than actual HR and MAP, to allow for physiological differences in heart rates and blood pressures between dogs of different breeds and sizes as well as individual physiological variability.

Differences in cage demeanour and sedation scores, age, proportion of shelter-owned versus privately owned dogs, weight, duration of anaesthesia, the length of the time between administration of the treatment (midazolam/placebo) and the end of anaesthesia, and the amount of propofol required for anaesthetic induction were investigated utilizing the Mann-Whitney U test, the Kruskal-Wallis H test, the chi square test or the independent-samples t-test as appropriate.

A two-way mixed ANOVA was used to examine variations in percentage change of HR from baseline, percentage change of MAP from baseline, PE'CO_2_, *f*_R_ and FE'Iso. The chi-square test of homogeneity, Fisher’s exact test, the Mann-Whitney U test and the Kruskal-Wallis H test were utilized as appropriate to assess group differences in anaesthetic complications and the treatment of those anaesthetic-related complications.

## Results

For the purpose of the study, 39 female dogs were recruited and randomly assigned to group P (20 dogs) or group M (19 dogs). The statistical significance level was set at α = 0.05 for all tests.

Breeds represented in our study were mixed breeds (14), Siberian Husky (9), Labrador (5), Staffordshire Bullterrier (2), Jack Russell Terrier (2), Cocker Spaniel (1), Samoyed (1), Shetland Sheepdog (1), Akita Inu (1), Golden Retriever (1), and German Shepherd (1). Mixed breeds (35.9%) and the Siberian Husky (23.1%) were overrepresented in our study population. In group P 13 animals were shelter-owned and 7 privately owned, in group M 11 animals were shelter-owned and 8 privately owned. No differences in the proportion of shelter-owned versus privately owned animals could be observed between groups and no differences in age, weight, cage demeanour or sedation score were detected (Table 3). Length of anaesthesia, the length of the time between administration of treatment (midazolam/placebo) and end of anaesthesia, and the amount of propofol required for anaesthetic induction did not differ between groups (Table 3).

**Table 2:** Sedation scoring system

Score	Description
1	Not very/not sedated: Able to stand up and walk. Fully responsive. No signs of depression, drowsiness, ataxia or altered character with respect to how it was without any medication
2	Slightly sedated: Able to stand up and walk. Fully responsive but slow to react. Mild signs of depression, drowsiness, ataxia or mild changes in character
3	Sedated: Able to stand up but reluctant to walk. Slow reaction to stimuli. Signs of depression, drowsiness, ataxia and changed character
4	Deeply/very sedated: Unable to stand up and walk. Unresponsive to stimuli. Depressed, drowsy and sleepy

Sedation scoring system used to assess 39 healthy female dogs admitted for elective ovariohysterectomy, following pre-medication with pethidine (3 mg kg^− 1^) and acepromazine (30 μg kg^− 1^) intramuscularly [21]

**Table 3:** Age, weight, cage demeanour and sedation scores, duration of anaesthesia, time from administration of midazolam/placebo to end of anaesthesia, and amount of propofol administered for anaesthetic induction

Parameter	Group P	Group M
Age (months)	17 (6–48)	11 (6–54)
Weight (kg)	19.0 ± 6.5	18.6 ± 7.3
Cage demeanour score	5 (2–5)	5 (2–5)
Sedation score	3 (2–4)	3 (2–3)
Duration of anaesthesia (min)	158.6 ± 25.1	160.8 ± 26.8
Time from administration of M/P to end of GA (min)	92.2 ± 20.2	95.5 ± 18.6
Amount of propofol administered for anaesthetic induction (mg kg^− 1^)	3.65 (2.5–4.6)	3.6 (2.4–6.7)

Age, weight, cage demeanour score, sedation score, duration of anaesthesia, time from administration of M/P to end of anaesthesia, and amount of propofol administered for anaesthetic induction in 39 healthy female dogs admitted for elective ovariohysterectomy. GA, general anaesthesia, M/P, Midazolam/Placebo. Group M: midazolam (0.25 mg kg^− 1^), group P: Hartmann’s solution (0.125 ml ^− 1^). Data presented as mean ± standard deviation or median (range) as appropriate

No statistically significant difference could be detected between groups for changes in percentage change in HR from baseline (*p* = 0.262) or percentage change in MAP from baseline (*p* = 0.078) (Table 4). For both percentage change in heart rate and blood pressure the main effect of time showed statistically significant differences (*p* = 0.0005 and p = 0.0005, respectively) at different time periods. For heart rate, the percentage change was statistically significantly higher for time period 5, compared to all other time periods. For the percentage change in blood pressure, time period 1 (baseline) and 2 were statistically significantly lower, compared to time periods 3, 4 and 5. Time period 1 was statistically significantly lower than time period 2. No statistical significant difference existed between time period 3 and 4, or between time period 4 and 5. Time period 3 was statistically significantly higher than time periods 1, 2 and 5. A statistical significant difference (*p* = 0.004) was detected in FE’Iso between group P and group M with group M requiring a significantly less FE’Iso (14.3%) than group P in the periods 3, 4, and 5, following the administration of midazolam (Table 4).

**Table 4:** Heart rate, mean arterial blood pressure, respiratory rate, end-tidal carbon dioxide, and end-tidal isoflurane concentration

Para-meter	Group	Period 1	Period 2	Period 3	Period 4	Period 5
HR	M	97.0 ± 12.3	104 ± 15.2	106 ± 11.9	105 ± 11.4	114 ± 15.9
	P	101 ± 18.9	99 ± 14.2	105 ± 12.1	106 ± 14.5	119 ± 15.8
% change in HR from baseline (%)	M	N/A	9.3 ± 17.3	11.2 ± 17.8	9.9 ± 20.9	18.1 ± 23.4
	P	N/A	−1.5 ± 12.6	4.7 ± 20.5	5.1 ± 27.0	19.6 ± 31.1
MAP (mmHg)	M	63.0 ± 6.7	72.0 ± 11.0	89.3 ± 9.5	86.6 ± 10.6	82.1 ± 13.2
	P	72.0 ± 10.1	81.1 ± 10.6	97.5 ± 12.5	90.8 ± 14.2	89.9 ± 10.7
% change in MAP from baseline (%)	M	N/A	11.0 ± 20.4	40.8 ± 18.4	39.0 ± 23.1	30.8 ± 23.9
	P	N/A	16.6 ± 17.2	38.2 ± 20.3	26.8 ± 19.8	27.9 ± 21.4
*f*_R_	M	16.0 ± 6.5	17.1 ± 7.8	13.1 ± 5.3	13.1 ± 5.1	14.7 ± 5.8
	P	19.0 ± 9.9	17.8 ± 10.8	17.5 ± 5.8	14.7 ± 6.0	15.3 ± 6.7
PE’CO_2_ (kPa)	M	5.11 ± 0.41	5.42 ± 0.46	6.19 ± 0.56	5.88 ± 0.36	5.66 ± 0.51
	P	5.35 ± 0.45	5.57 ± 0.56	5.91 ± 0.72	5.86 ± 0.7	5.76 ± 0.62
FE’Iso (%)	M	1.03 ± 0.14	1.15 ± 0.11	1.2 ± 0.17*	1.22 ± 0.2*	1.23 ± 0.1*
	P	1.05 ± 0.11	1.16 ± 0.11	1.35 ± 0.15	1.43 ± 0.16	1.42 ± 0.19
Difference FE’Iso (%)	M/P	9.1	8.3	14.3	14.3	14.3

Mean values for heart rate (HR), mean arterial blood pressure (MAP), end-tidal isoflurane concentration (FE’Iso); respiratory rate (*f*_R_); and end-tidal carbon dioxide (PE’CO_2_), and difference between groups in FE’Iso expressed in percent (% difference FE’Iso) for group P and group M for each time period (1–5). M/P, Midazolam/Placebo; %, percentage; Group M: midazolam (0.25 mg kg^− 1^), group P: Hartmann’s solution (0.125 ml kg^− 1^). Period 1 (baseline): time in operating room until start of surgery; Period 2: start of surgery to start of ovarian manipulation; Period 3: manipulation of the ovaries; Period 4: manipulation of the uterus; and Period 5: abdominal closure. N/A, not applicable. Data presented as mean ± standard deviation. *Statistical significance (*p* < 0.05)

Rescue analgesia was required intra-operatively in four dogs: three dogs in group P and one dog in group M. Post-operatively the dog from group M and one of the dogs from group P required additional rescue analgesia. Additionally, another dog from group P and three more dogs from group M required rescue analgesia post-operatively. Neither intra-operative (*p* = 0.605) nor post-operative (*p* = 0.407) rescue analgesia requirements displayed a statistically significant difference between groups. Sedation for recovery due to dysphoria was required for two dogs in group M, but for no dog in group P, with no statistical significance shown between groups (*p* = 0.231).

Five dogs in group M and seven dogs in group P required mechanical ventilation. No statistical difference in *f*_R_ (*p* = 0.533), PE’CO_2_ (*p* = 0.067) or the need for mechanical ventilation (*p* = 0.731) was detected between groups (Table 4).

Adverse events during anaesthesia included hypotension (group P: 13 dogs; group M: 17 dogs), hypertension (group P: four dogs; group M: two dogs), bradycardia (group P: two dogs; group M: 1 dog), tachycardia (group P: five dogs; group M: three dogs), apnoea (group P: two dogs; group M: three dogs), hypercarbia (group P: five dogs; group M: seven dogs) and hypothermia (group P: three dogs; group M: five dogs). No statistically significant difference in the incidence of complications in group M and group P could be detected.

A large number of dogs required bolus fluid therapy, due to intra-anaesthetic hypotension. Prior to treatment with midazolam or placebo, dogs in group M required statistically more bolus fluid therapy with succinylated gelatine solution than dogs in group P (*p* = 0.012). However, no statistical difference was observed for crystalloid fluid bolus pre-treatment, or with colloid or crystalloid fluid bolus post study drug administration.

Three dogs in group M also received dobutamine CRI, and, of those three dogs, two additionally received noradrenaline CRI to treat hypotension. One dog in group M and two dogs in group P received anticholinergics. The dog in group M and one dog in group P were administered glycopyrrolate. The other dog in group P received atropine. No statistical significance could be demonstrated for the use of dobutamine CRI, noradrenaline CRI or anticholinergic agents.

## Discussion

A significant reduction in isoflurane requirement was detected in healthy dogs undergoing elective ovariohysterectomy after the administration of midazolam intravenously. However, the results of the presented study show no significant changes in recorded cardiovascular or respiratory parameters between group P and group M during surgical removal of the ovaries.

Several studies have investigated the MAC-sparing effect of midazolam on volatile anaesthetics in humans [23], goats [24, 25], and dogs [16, 26–28]. In 1988 Hall et al. demonstrated an enflurane MAC-sparing effect of midazolam CRI in dogs using the tail clamp technique. Takeuchi et al. (1991) detected a ‘significant and proportional decrease’ in the MAC of isoflurane, determined via tail clamping, in dogs receiving a midazolam CRI. Additionally, the study investigated the effect exerted by midazolam CRI together with isoflurane (1 MAC) on cardiovascular parameters during a period without any stimulation and detected no differences with and without midazolam. A study by Stegmann et al. exploring the effect of midazolam premedication (0.1 mg kg^-1^) in propofol-induced and isoflurane-maintained dogs undergoing ovariohysterectomy also found a reduction in requirement of isoflurane with midazolam administration [28]. In their study, the minimum isoflurane concentration required was determined by testing for a pedal reflex. However, Seddighi et al. (2011), when studying the influence of midazolam on the MAC of isoflurane in dogs using electrical stimulation over the mid-ulnar area, only observed a clinically significant MAC reduction at infusion rates of 10 μg kg^− 1^ min^− 1^ with a loading dose of 0.8 mg kg^− 1^ [16]. At lower, clinically appropriate, midazolam doses (0.2 mg/kg IV and 0.4 mg kg^-1^) followed by a continuous rate infusion (CRI) of 2.5 μg/kg/min and 5 μg kg^-1^ min^− 1^, respectively, a decrease in MAC of isoflurane by only 11 ± 5% was observed [16]. In our study, a 14.3% decrease in isoflurane requirement was detected after administration of midazolam in comparison to the placebo group. The authors considered this to be a modest clinically significant difference in end-tidal anaesthetic concentration.

Midazolam is mainly used for its anticonvulsant, sedative and hypnotic, and muscle relaxant effects in veterinary medicine [15]. An antinociceptive action is generally not ascribed to systemic administration of midazolam in dogs [16]. The effect of systemic administration of opioids and benzodiazepines combined has been investigated in rats [29], mice [30] and humans [31, 32]. A positive analgesic effect was observed in human patients after administration of flumazenil, a benzodiazepine antagonist, in both patients that had been administered diazepam as part of the anaesthetic protocol as well as in patients without prior benzodiazepine administration [31, 32]. The negative analgesic interaction between opioids and benzodiazepine agonists is thought to be caused by a modulation of neuronal pathways via both GABA_A_ [31, 33] and NMDA receptor mechanisms [34, 35]. To these authors’ knowledge the negative impact of benzodiazepines on postoperative opioid requirements has not been investigated in a clinical veterinary setting, but in our dogs no negative effect was observed.

Approximately 77% of our study population, irrespective of the group assignment, became hypotensive post-induction of anaesthesia. Hypotension is a common complication in human and veterinary anaesthesia [36, 37] and is particularly prevalent between induction of anaesthesia and the beginning of surgery due to the influence of anaesthetic drugs in a period of very little noxious stimulation [36].

The phenothiazine acepromazine, a commonly used sedative, causes a decrease in blood pressure at clinically relevant doses [38]. Its vasodilatory action is mediated via α_1_ adrenoceptor antagonism, depression of central vasomotor centres and direct action on vascular smooth muscle [39, 40]. Anaesthesia was induced with the short-acting injectable anaesthetic propofol, which can lead to hypotension through inhibition of the sympathetic nervous system, impairment of baroreflex regulatory mechanisms, and reduction in vascular tone through a direct action on veins and arterioles [36, 41, 42]. Anaesthetic maintenance with volatile agents, such as isoflurane, causes dose-dependent myocardial depression associated with a decline in stroke volume as well as a decrease in systemic vascular resistance, resulting in a reduction in arterial blood pressure [43–45].

Opioids play a key role in the management of peri-operative analgesia and sedation, often as a part of a multimodal approach [46, 47]. The cardiovascular effects of opioids at clinically relevant doses are generally minimal [48, 49], although they can be associated with a centrally mediated bradycardia [50]. With mild bradycardia, cardiac output is maintained through an increase in stroke volume, but more severe decreases in heart rate will result in a reduction in cardiac output [51]. Unfortunately, the combination with anaesthetic drugs, including benzodiazepines, enhances the negative cardiovascular effects of opioids [52, 53]. Both morphine and pethidine are commonly used full μ agonist opioid drugs, but pethidine, unlike morphine, is unlikely to cause nausea and vomiting, when administered as part of the premedication [54]. The opioid doses administered in our study protocol are at the lower end of recommended dose ranges and we did not observe any negative cardiovascular effects immediately associated with the administration of the opioids themselves or when administering midazolam intra-operatively.

Our study was designed to investigate the effect of midazolam, and all dogs received an otherwise uniform anaesthetic protocol. It is therefore impossible to deduce how much each individual drug administered, other than midazolam, contributed to the overall observed hypotension.

Final year veterinary students are required to perform one elective ovariohysterectomy or castration under direct supervision by an experienced surgeon at our institution. Ovariohysterectomy performed by final year students have been observed to take twice as long as those performed by experienced surgeons [55–57]. This held true for our study population, resulting not only in a very long overall duration of anaesthesia, but also in a particularly prolonged period inside the operating theatre without stimulation of the animal, while students were getting ready to start the surgery.

Dogs in group M received significantly more colloid fluid therapy in time periods 1 and 2 for the treatment of hypotension, but this finding was not linked to group difference in heart rate or blood pressure during the same time periods. It can be speculated, that dogs allocated to group M incidentally were more prone to hypotension intra-operatively and required more fluid bolus therapy to compensate for this fact. Independent of this finding, the administration of midazolam did not have a significant effect on blood pressure or heart rate in the dogs studied here.

Blood pressure significantly increased with the start of the manipulation of the ovaries in both groups and did not significantly decrease during the following time periods. This is similar to findings in other studies [58, 59]. Traction on the suspensory ligament of each ovary and ligation of the ovarian pedicle have been previously identified as the most stimulating aspects of an ovariohysterectomy, as blood pressure increases significantly during this phase and signs of inadequate depth of anaesthesia are often observed [60, 61]. Surprisingly, heart rate was higher during the time period of abdominal closure than during any other time period. Abdominal wall closure appears to exert a major surgical stimulus. The abdominal wall is richly innervated by branches of the spinal nerves T7 – L3 and loco-regional anaesthesia in the form of a transverse abdominis plane (TAP) block has been shown to be an effective analgesic adjunct for abdominal surgery in cats and dogs [62–64].

Dose-dependent respiratory depression, characterised by an increase in arterial partial pressure of carbon dioxide, is an important side effect of many anaesthetic drugs. Opioids elicit respiratory depression by acting on μ and δ opioid receptors in medullary respiratory centres and on μ opioid receptors at chemoreceptor sites [65]. Benzodiazepines act mainly on GABA_A_ receptors. High concentrations of GABA_A_ receptors can be found in the dorsal and ventral medullary groups and are thought to have an important role in the control of ventilation [65]. Benzodiazepines and μ agonist opioids in combination were shown to cause a more severe respiratory depression than either drug group alone in humans [66–68]. Despite extensive research the exact mechanism for the potentiation of respiratory depression observed with the combination of opioids and benzodiazepines remains unascertained. Whether opioids and benzodiazepines have simply an additive action, or if there is a more complicated, most likely multifactorial interaction at play involving pharmacokinetic or pharmacodynamic processes, remains to be proven [68, 69]. However, at the doses used in our study, no significant difference in respiratory depression between the two groups was evident.

No pre-anaesthetic haematological and serum biochemistry testing was performed and we cannot fully exclude the presence of underlying disease in the dogs enrolled in the study. Hepatic or renal disease could negatively affect the metabolism and elimination of midazolam and other drugs. A delayed drug clearance would be expected to result in a prolonged duration of action, and possibly more severe drug effects, which was not clinically observed. Moreover, studies in humans [70] and dogs [71, 72] have detected little benefit of routine pre-anaesthetic blood testing in young healthy individuals unless history or physical examination results were indicative of potential health problems.

While the number of dogs recruited for this study is appropriate according to the performed power analysis, it is a small sample size from a statistical point of view and many of the observed adverse events occurred at a very low frequency in our study population. The significance of statistical results inferred from small frequency events and/or small sample sizes should be critically examined [73, 74] and future studies with larger sample sizes would be desirable.

Due to the clinical nature of the study, inspired isoflurane concentration was adjusted intra-operatively to maintain an appropriate level of anaesthetic depth, preventing any untoward effects of inadvertent anaesthetic overdose. This resulted in the above described and discussed isoflurane-sparing effect observed with the administration of midazolam. It is possible that, if the inspired isoflurane concentration had not been altered, differences between groups in cardiovascular parameters could have been detected.

## Conclusion

In conclusion, a modest isoflurane-sparing effect (14.3%), was observed with the administration of a single bolus of midazolam in dogs prior to manipulation of the ovaries during ovariohysterectomy in our clinical study population. The findings of this study do not support the use of midazolam to attenuate cardiovascular responses in dogs undergoing elective ovariohysterectomy.
